# Effect of forward and backward sloped support surfaces on postural equilibrium and ankle muscles activity

**DOI:** 10.1371/journal.pone.0305840

**Published:** 2024-06-27

**Authors:** Siripatra Atsawakaewmongkhon, Annabelle Couillandre, Alain Hamaoui

**Affiliations:** 1 CIAMS, Université Paris Saclay, Orsay, France; 2 CIAMS and SAPRéM, Université d’Orléans, Orléans, France; Universita Politecnica delle Marche Facolta di Ingegneria, ITALY

## Abstract

**Introduction:**

Although sloped surfaces are common in daily living, most studies of body balance are carried out on flat surfaces, and few data are available for sloping angles below 14°.

**Objectives:**

The purpose of this study was to explore the effect of forward and backward sloping surfaces at 7° and 15° on postural equilibrium and the activity of flexor/extensor ankle muscles.

**Methods:**

Fifteen healthy subjects (8 males and 7 females) (27.67 ± 3.9 years) underwent a posturographic examination associated with a surface electromyogram (EMG) of tibialis anterior (TA), soleus (Sol) and gastrocnemius medialis (GasM) under five conditions of support inclination: 0° (H0), backward inclination at 7° and 15° (DF7 and DF15), forward inclination at 7° and 15° (PF7 and PF15).

**Results:**

Results showed that the center of pressure (CP) was shifted according to the surface slope, with a forward move in PF7 (p <0.001) and PF15 (p <0.001) and a backward move in DF7 (p <0.01) and in DF15 (p <0.001). The mean displacement of the CP along the anterior-posterior axis (Xm) was increased in DF15 (p <0.01) relative to the H0 condition but reduced in PF7 (p <0.01). The normalized EMG revealed higher values when the muscles were in a shortened position (PF7 for Sol, p <0.05; PF15 for GasM, p <0.01; DF15 for TA, p<0.01) and lower values of GasM and Sol when lengthened (DF15, p <0.05).

**Conclusion:**

Our findings indicate that standing on a backward sloped surface impairs body balance, while low-angle forward sloped surfaces might improve postural stability. Muscular activity variations of the ankle flexors/extensors, which are stretched or shortened, also seem to be related to the length-tension relationship of skeletal muscles.

## Introduction

The control of postural balance is a prerequisite for most motor tasks in daily life. According to Gurfinkel et al. (1995) [1], it is ensured by at least two mechanisms. The first mechanism sets the activity of anti-gravity postural muscles in a given configuration of the multi-link kinematic chain (conservative system), while the other stabilizes this position (operative system). The conservative system requires the activity of a large number of postural muscles at a low level, mainly below 10% of the Maximum Voluntary Contraction (MVC); the only exception is the soleus, which can reach 20% of the MVC [2]. The operative system is assumed to involve ankle and hip mobility via the mechanisms of hip and ankle strategies described by Horak and Macpherson (1996) [3]. In addition, postural control involves the mobility of a large part of the postural chain in relation to the respiratory disturbance to posture [4]; however, various studies have considered that postural sway mainly occurs around the ankle axis under the principle of an inverted pendulum model [5, 6].

Sloped surfaces are common in daily living, as when the ground surface is inclined or elevated to reduce the potential risk of urban flood [7]. These surfaces are also found in access ramps for people with disabilities and specific professional activities such as roofing. In those conditions, maintaining a stable standing body balance, with the lower limbs and trunk lined up along a common vertical axis, would require an adaptation at the ankle level, including changes in angular position and muscle tension. An articular position that is less neutral would offer reduced articular free play for the operative system. An imbalance of the passive tension between the agonist/antagonist muscles controlling ankle flexion/extension might also impair the ability to stabilize this joint. As expected, posturographic studies have described an impaired body balance while standing on sloped surfaces [8–14]. Some studies using surface electromyogram (EMG) examination have shown an increased activity of the soleus and the gastrocnemius while the feet are in plantar flexion [8, 13, 15]. A higher activity of the tibialis anterior associated with a dorsiflexion of the feet has also been described [8, 16], but not systematically [13, 15]. It should also be noted that most of these studies used high-sloping angles greater than or equal to 14°, which are often encountered in roofing [9, 11–14, 16]. A few studies use 10° as the minimum value [8, 15], yet this angle is greater than the usual walkway slope, whose standard is at 4.76° [17]. Additionally, only two studies have combined posturographic and EMG examinations [8, 13]. Neither has concurrently assessed the EMG of soleus and gastrocnemius, even though they present a different activity level while standing [2]. There is also a lack of data on the mean position of the center of pressure (CP), which is also likely to vary with the surface slope. All these variations may have implications in assessing the risks of falling when using slope surfaces encountered in daily living. Their understanding must also help improve accessibility standards and implement rehabilitation programs to reinforce the autonomy of people suffering from physical disabilities.

This study aims to assess the effect of sloped surfaces encountered in daily living on body balance and activity level of the three main flexor/extensor muscles (soleus, gastrocnemius medialis and tibialis anterior). The central hypothesis is that maintaining posture on a sloped surface induces a decreased postural stability associated with an activity variation of the stretched and shortened muscles. This study is qualified as an experimental study. The experimental design includes posturographic and surface EMG examination while standing with different inclinations of the support surface in the sagittal plane (-15°, -7°, 0°, 7°,15°).

## Materials and methods

### Subjects

Fifteen healthy adults (8 males and 7 females, all right-handed; age: 27.67 ± 3.9 years old; weight: 61.47 ± 9.99 kg; height: 168.2 ± 7.70 cm; body mass index: 21.57 ± 1.88 kg/m^2^), participated in the experiment. They were recruited using posters and flyers. The sample size, calculated using G*Power software, equaled 14. All subjects were free of any pathology or medical treatment that might affect balance control or ankle joint mobility. No alcohol consumption was allowed for the twelve hours prior to the experiment. The experimental protocol was approved by the local ethics committee, CER Paris-Saclay, with the reference CER-Paris-Saclay-2022-045. Written informed consent was obtained from each participant in accordance with the declaration of Helsinki. The experiments started on the 10^th^ September, 2022, and ended on the 6^th^ October, 2022.

### Materials

#### Force platform and slant board

A six-channel force plate (AMTI-OR6, 60 x 60 x 12.57 cm), which collected the ground-reaction forces and moments applied at its top surface, was used to calculate the coordinates of the CP along the anterior-posterior and the medial-lateral axes.

**Fig 1:**
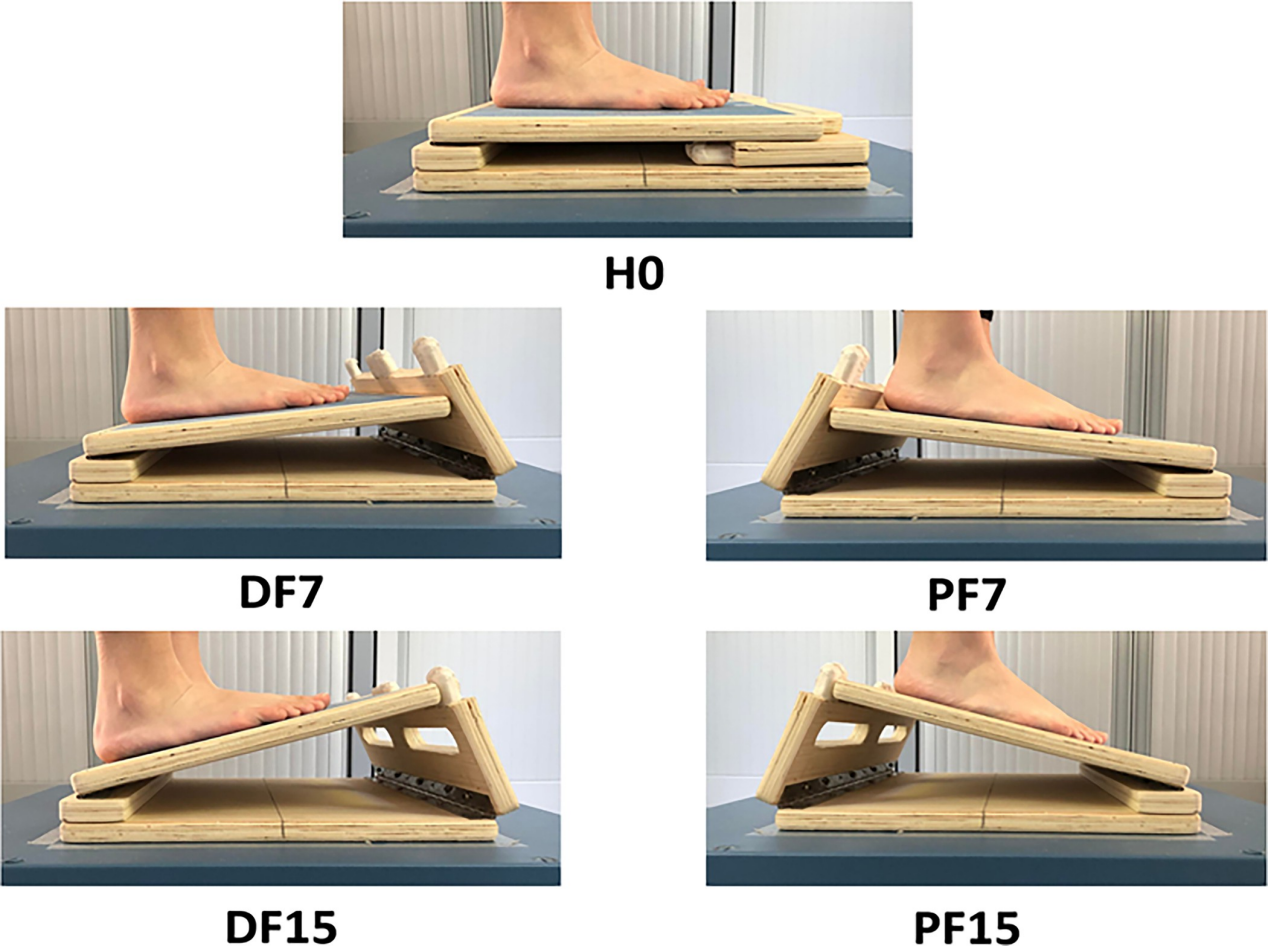
Slant board mounted on the force plate in all different experimental conditions. Horizontal (H0, reference condition), dorsal flexion at 7° (DF7) and 15° (DF15), plantar flexion at 7° (PF7) and 15° (PF15).

An adjustable and customized slant board (43 x 33 x 5.5 cm) set the support surface in the sagittal plane at three different sloping levels (0°, 7°, and 15°). It was used in five different experimental conditions: horizontal (H0, reference condition), dorsal flexion at 7° (DF7) and 15° (DF15), and plantar flexion at 7° (PF7) and 15° (PF15) (Fig 1). The board was centered and fixed on the force plate with adhesive gum and had a 180° turn to switch from inclined to declined conditions. It was made of wood and covered with sandpaper to prevent slipping. Sloping levels have been chosen at 7° and 15° to allow for comparisons with existing studies using values greater or equal to 10° [8–13, 15] and also to test a condition closer to the lower values encountered in daily living [17]. 15° was chosen as the maximum value because it is close to the range of motion of the ankle in dorsal flexion [18] and should allow an almost complete compensation of the slope at the ankle level.

The coordinates of the CP are calculated from the following formulas:

$$x_{p}=\frac{-hF_{x}+M_{y}}{F_{z}}$$

$$y_{p}=\frac{-hF_{y}-M_{x}}{F_{z}}$$

with

- *X*_*p*_: CP along the anterior-posterior axis;
- *Y*_*p*_: CP along the medial-lateral axis;
- *h*: distance between the center of the slant board and the origin of the force plate;
- *F*_*x*_: reaction force of the ground along the anterior-posterior axis;
- *F*_*y*_: reaction force of the ground along the medial-lateral axis;
- *F*_*z*_: ground reaction force along the vertical axis;
- *M*_*x*_: moment along the anterior-posterior axis;
- *M*_*y*_: moment along the medial-lateral axis.

#### Surface electromyogram (sEMG)

**Fig 2:**
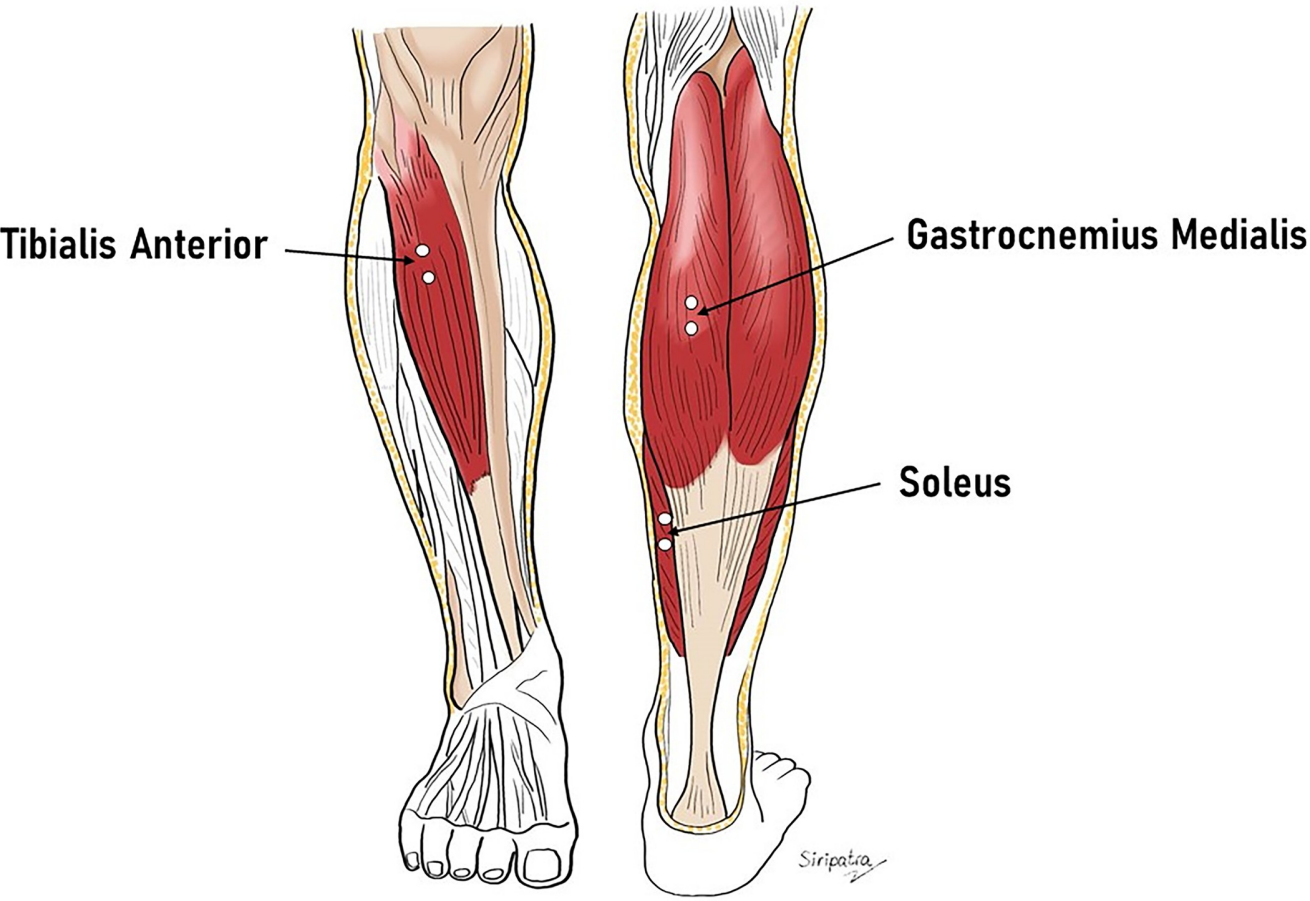
EMG electrodes placement of the three tested muscles.

A 16-channel wireless EMG device (Pico EMG model, Cometa, Milan, Italy) was used to quantify the normalized surface electrical activities of the main flexor/extensor of the ankle and focused on tibialis anterior (TA), gastrocnemius medialis (GasM) and soleus (Sol). The participants’ skin was shaved where needed, abraded, and cleaned with alcohol to reduce skin impedance to below 5kΩ. 10-mm diameter (conductive area) Ag/AgCl pre-gelled disposable surface electrodes (H124SG KendallTM) were applied in a bipolar configuration over the muscle belly parallel to the direction of the muscle fibers on the dominant side of the body. The inter-electrode distance was 20mm for all sites. In accordance with Barbero et al. (2012) [19], the electrode placement was as follows: Sol: on the lower 1/3 of the line connecting the medial condyle of the femur to the medial malleolus; GasM: on the most prominent part of the GasM muscle body; TA: on the upper 1/3 of the line connecting the medial malleolus to the head of the fibula, (Fig 2). All electrode placements were validated using palpation and manual resistance tests based on SENIAM recommendations [20]. More in detail, palpation tests confirmed that the electrodes were effectively located under the muscle belly, and a series of resisted contractions/relaxation were used to check that the EMG signal varied according to muscle activity.

To allow for normalization of the EMG signals by their maximum values, two 3-s isometric MVCs were first carried out for each muscle, with an inter-trial 30-s resting time. The TA and GasM MVC tests were performed in a supine lying posture with knees extended. The subject had to exert dorsiflexion (TA) and plantar flexion (GasM) against the experimenter’s resistance force. For the Sol, the subject was lying prone with the knee flexed at 90° on the active side and had to exert a plantar flexion against the experimenter. This conditions were chosen with respect to the main function of each muscle and according to the "muscle testing" method " [21]. EMG recordings were then taken during all posturographic tests.

#### Data acquisition system

Data from the force plate and the EMG system were digitized at 1000 Hz using a CompactDAQ with 9215 modules (National Instrument, Austin, USA), controlled by a custom program written with LabVIEW software (National Instrument, Austin, USA).

### Procedure

**Fig 3:**
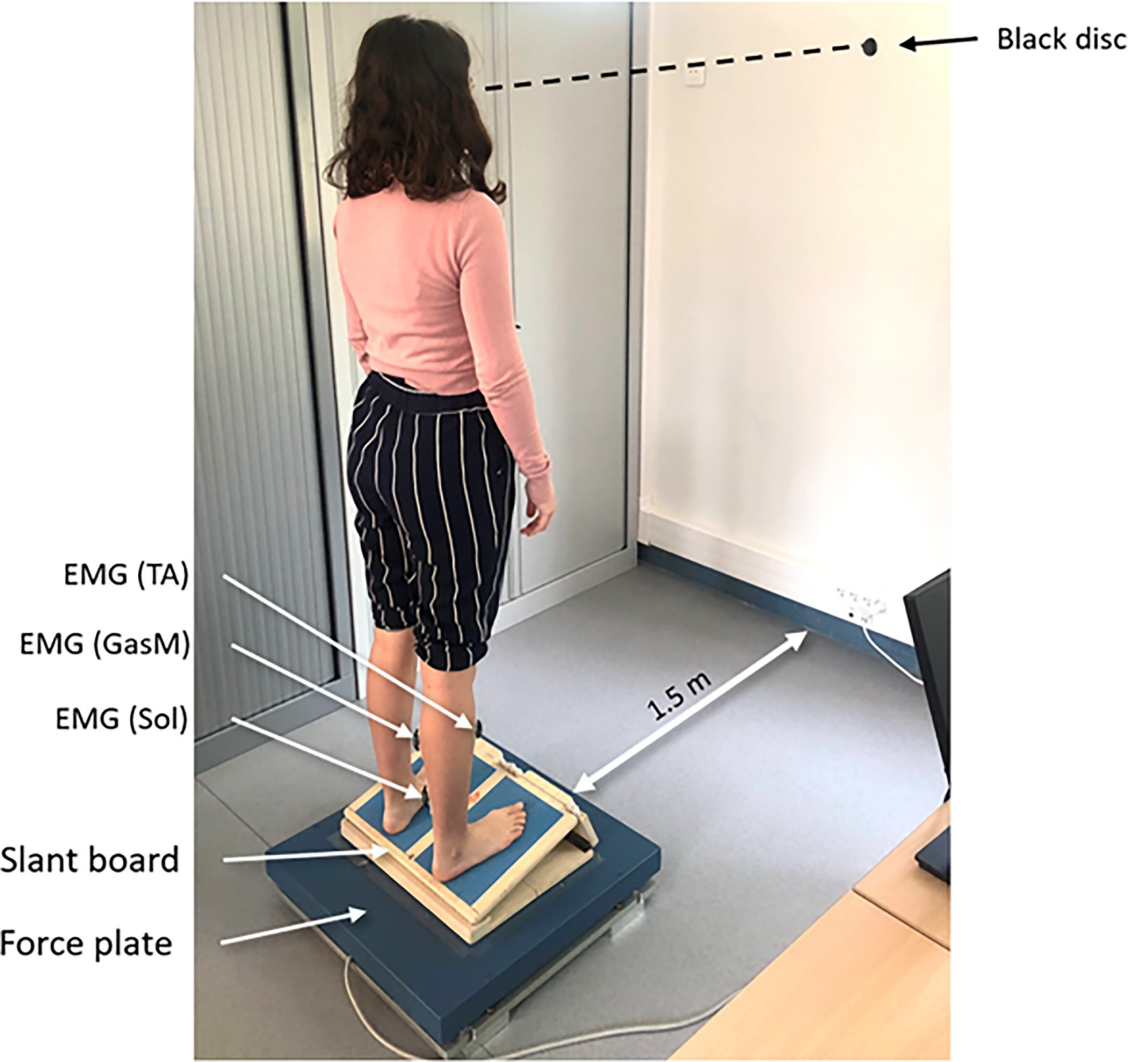
Subject equipped with EMG electrodes standing on the slant board in a DF15 condition.

The experiment started with a 2-min warm-up session with light exercises involving the whole body. Subjects stood barefoot on the slant board in their natural posture, with their arms hanging at the sides and their feet apart at hip width. They were instructed to move as little as possible and keep their gaze fixed on a 5-cm black disc placed at the subject’s eye level on a wall located 1.5m in front of them (Fig 3). The recording started a few seconds after a "TOP" signal indicated the beginning of the trial, and its end was associated with an electronic beep. Four trials of 30s each were performed in five experimental conditions varying the support surface slope: horizontal 0° (H0), inclined (toes up) at 7° (DF7) and 15° (DF15), declined (toes down) at 7° (PF7) and 15° (PF15). After a training period during which the subject became familiar with the five conditions, the recording started with condition H0, the reference posture. The other conditions were randomly assigned to avoid any order effect. A rest time of 20s between trials and 2min between conditions was given to prevent fatigue.

### Data analysis

#### Posturographic data

Five classical posturographic indices assessing mean position, mean deviation, and mean velocity were used to assess body balance:

- X0: mean position of the CP along the anterior-posterior axis
- Y0: mean position of the CP along the medial-lateral axis
- Xm: mean deviation of the CP along the anterior-posterior axis
- Ym: mean deviation of the CP along the medial-lateral axis
- Vm mean velocity of the CP in the horizontal plane

#### Normalized EMG

For each muscle, the mean rectified EMGs were calculated from the complete recordings, and the values were expressed as a percentage of the data obtained in the MVC condition.

Normalized EMG in posturographic condition = mean rectified EMG in MVC condition *100 / mean rectified EMG in posturographic condition.

### Statistical analysis

A one-way repeated-measures analysis of variance (ANOVA) was conducted with the support surface slope as a dependent variable presenting five modalities: H0, DF7, DF15, PF7, and PF15. When a statistical difference was reached, a simple contrast test was used with H0 as the reference condition. The significance level was set at p-values <0.05. All statistical analyses were performed using JASP Statistics software (version 0.18.3.0, The Netherlands).

## Results

### EMG data

**Fig 4:**
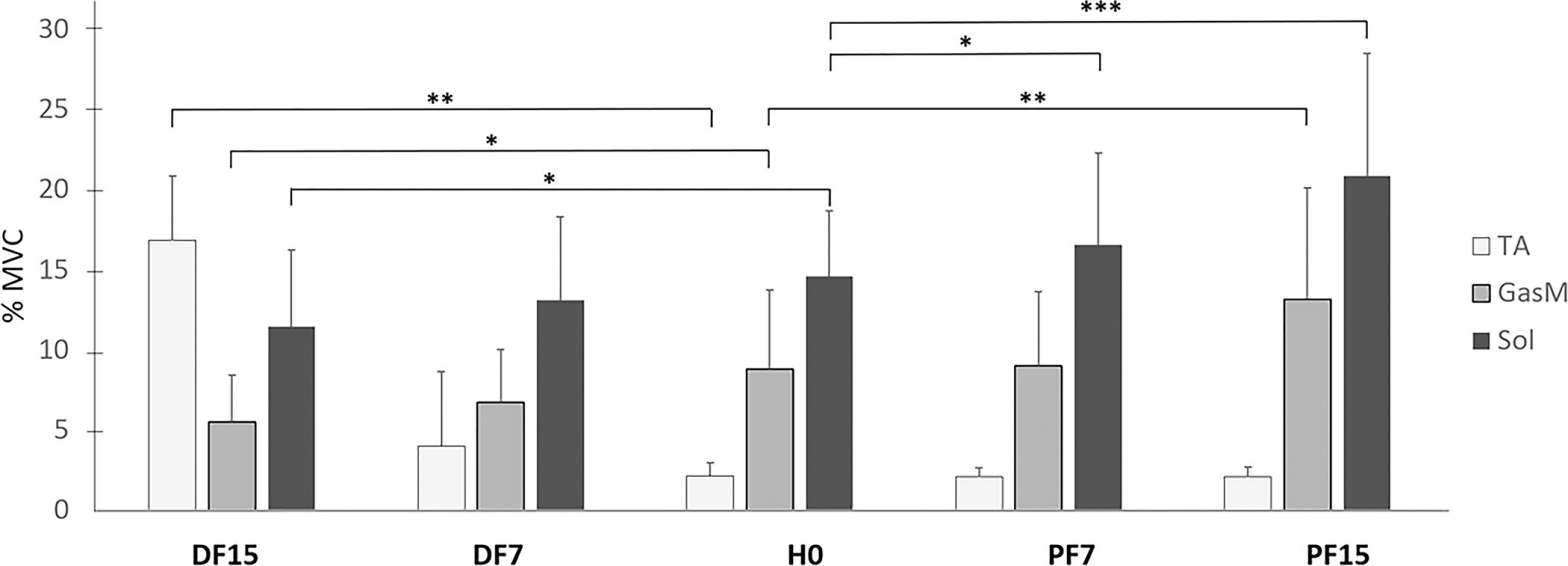
Normalized EMG (% maximal voluntary contraction, or MVC) as a function of surface slope. EMG of tibialis anterior (TA), soleus (Sol) and gastrocnemius medialis (GasM) are presented in each condition of surface slope: horizontal (H0), dorsal flexion at 7° (DF7) and 15° (DF15), and plantar flexion at 7° (PF7) and 15° (PF15), (*p <0.05, **p <0.01 and ***p <0.001).

**Fig 5:**
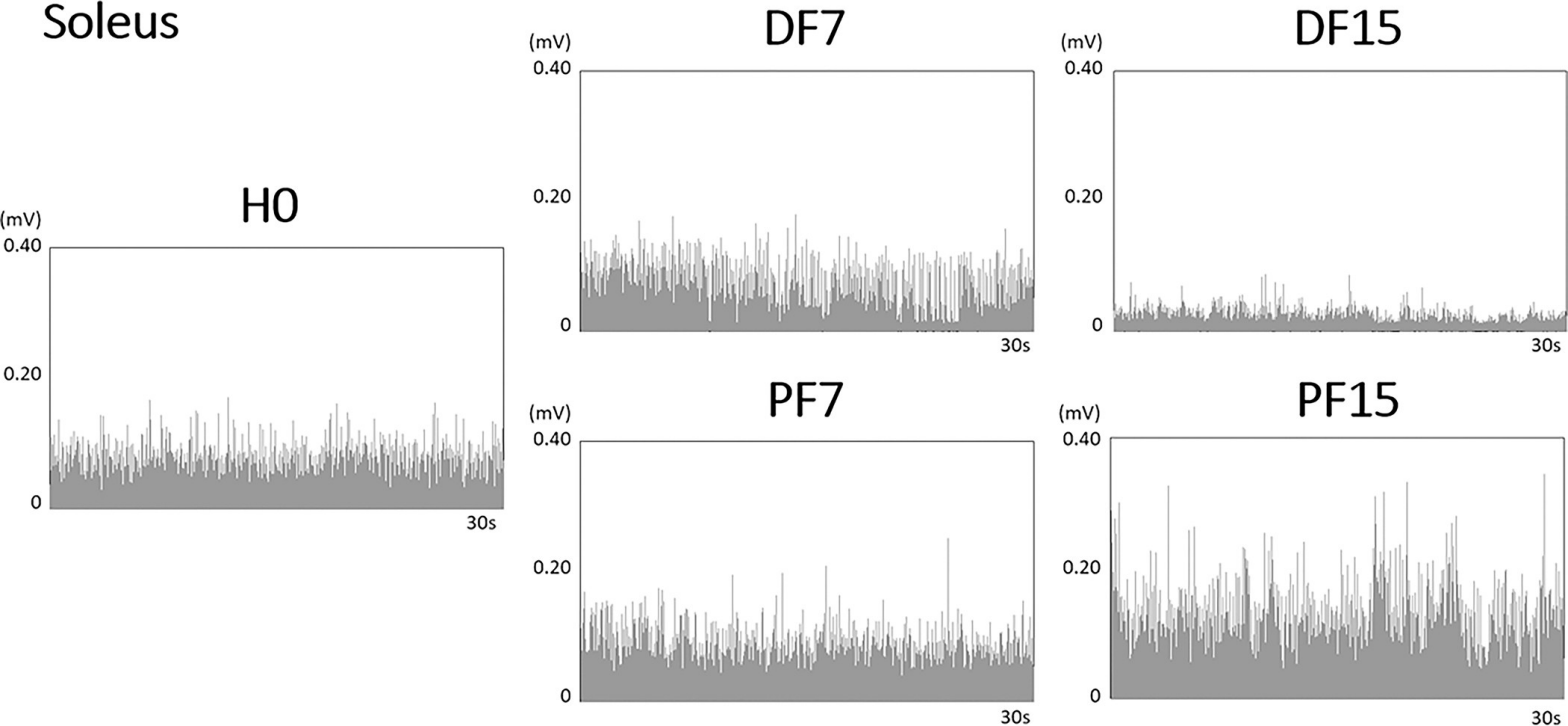
Representative traces of the 30-s rectified EMG signal of the soleus (Sol). Data are presented in mV in each surface slope condition: horizontal (H0), dorsal flexion at 7° (DF7) and 15° (DF15), and plantar flexion at 7° (PF7) and 15° (PF15).

The repeated measures ANOVA showed a significant effect of the surface slope on normalized EMG for all three muscles that were tested, with higher values for conditions where the muscle was shortened (Fig 4 and Table 1). More precisely, the contrast analysis revealed an increase between H0 and DF15 (p <0.01) for TA, an increase between H0 and PF15 (p <0.01) for GasM, and an increase between H0 and PF7 (p <0.05) and H0 and PF15 (p <0.001) for Sol. In addition, ankle extensors showed lower values for conditions in which they were lengthened, with a significant difference between H0 and DF15 for both GasM and Sol (p <0.05). These variations were visible in EMG raw data, as can be seen for Sol (Fig 5).

**Table 1:** Mean rectified EMG of tibialis anterior (TA), gastrocnemius medialis (GasM) and soleus (Sol) in each surface slope condition.

	TA (%MVC)	p	GasM (%MVC)	p	Sol (%MVC)	p
**H0**	(2.18 ± 0.81)		(8.81 ± 4.93)		(14.60 ± 4.10)	
**DF7**	(4.04 ± 4.61)	ns	(6.76 ± 3.27)	ns	(13.11 ± 5.22)	ns
**DF15**	(16.84 ± 4.04)	**	(5.51 ± 2.92)	*	(11.46 ± 4.80)	*
**PF7**	(2.09 ± 0.60)	ns	(9.02 ± 4.66)	ns	(16.56 ± 5.73)	*
**PF15**	(2.10 ± 0.60)	ns	(13.18 ± 6.95)	**	(20.87 ± 7.62)	***

Mean values ± standard deviations are expressed as a percentage of the mean value obtained in MVC condition. Slope conditions are: horizontal (H0), dorsal flexion at 7° (DF7) and 15° (DF15), and plantar flexion at 7° (PF7) and 15° (PF15). p-values of the simple contrast analysis, with H0 as the reference condition, are presented (*p <0.05, **p <0.01, ***p <0.001 and ns = non-significant).

### Posturographic data

**Fig 6:**
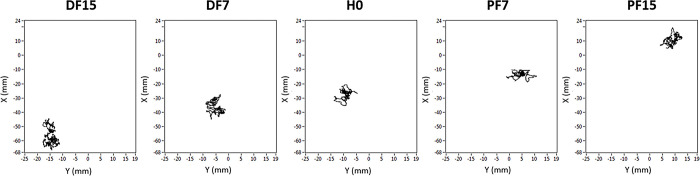
Representative traces of 30-s CP trajectory in each experimental condition. From left to right: dorsal flexion at 15° (DF15) and 7° (DF7), Horizontal (H0), and plantar flexion at 7° (PF7) and 15° (PF15). Y represents the medial-lateral axis, and X the anterior-posterior axis.

A visual inspection of CP trajectory suggests that the mean position of the CP is correlated with the surface slope direction and amplitude. In the H0 reference condition, the posturogram appeared forward in plantar flexion (PF7 and PF15) and backward in dorsal flexion (DF7 and DF15), with maximum deviations for the highest slope levels (DF15 and PF15) (Fig 6). The one-by-one inspection of the posturograms also revealed higher values of the global surface in sloped surface conditions but with no visible effect of direction (plantar flexion versus dorsal flexion) or amplitude (7° versus 15°) (Fig 6). The subsequent analysis of posturographic parameters that assess the mean position, deviation, and velocity confirmed this trend and highlighted more specific variations.

**Fig 7:**
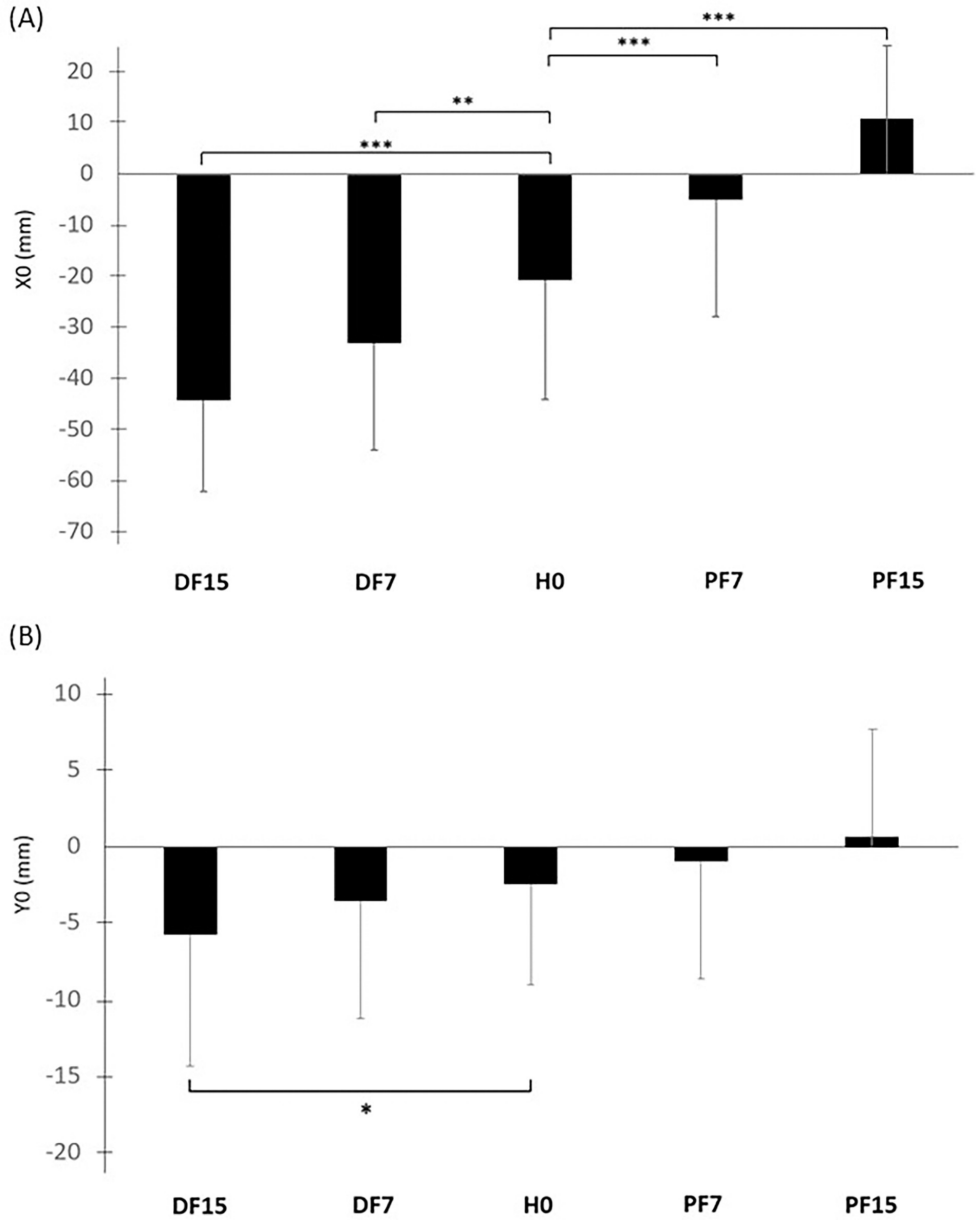
Mean position of CP along the anterior-posterior (X0) (A) medial-lateral (Y0) (B) axes as a function of surface slope. Means and standard deviations are presented for each experimental condition: horizontal (H0), dorsal flexion at 7° (DF7) and 15° (DF15), and plantar flexion at 7° (PF7) and 15° (PF15), (*p <0.05, **p <0.01 and ***p <0.001).

The analysis of variance revealed a global effect of surface slope on the average position of the CP along the anterior-posterior axis (X0) (p <0.001). The contrast analysis showed higher values in plantar flexion (p <0.001 in PF7 and p <0.001 in PF15) and lower values in dorsal flexion (p <0.01 in DF7 and p <0.001 in DF15) (Fig 7A and Table 2), with the lowest values in DF15 and the highest in PF15. In other words, the CP is shifted toward the direction of the support surface slope, backwards for inclined and forwards for declined. Along the medial-lateral axis (Y0) (Fig 7B and Table 2), a significant global effect was also observed (p <0.05). However, the contrast analysis only showed a significant decrease in DF15 relative to H0 (p <0.05). As the right side corresponds to negative values for the medial-lateral axis of the force plate, the CP was shifted to the right in DF15.

**Table 2:** Posturographic parameters as a function of surface slope.

	X0 (mm)	p	Y0 (mm)	p	Xm (mm)	p	Ym (mm)	p	Vm (mm/s)	p
**H0**	(-20.82 ± 23.43)		(-2.43 ± 6.60)		(2.83 ± 0.66)		(1.20 ± 0.44)		(378.16 ± 77.57)	
**DF7**	(-33.15 ± 20.95)	**	(-3.53 ± 7.70)	ns	(3.06 ± 0.86)	ns	(1.17 ± 0.41)	ns	(384.86 ± 80.13)	**
**DF15**	(-44.43 ± 17.82)	***	(-5.79 ± 8.55)	*	(4.11 ± 1.51)	**	(1.46 ± 0.38)	*	(391.84 ± 79.06)	**
**PF7**	(-5.16 ± 22.89)	***	(-0.97 ± 7.72)	ns	(2.25 ± 0.51)	**	(1.27 ± 0.51)	ns	(376.64 ± 77.78)	ns
**PF15**	(10.73 ± 25.08)	***	(0.69 ± 7.67)	ns	(2.77 ± 0.94)	ns	(1.31 ± 0.34)	ns	(385.71 ± 71.86)	ns

Mean values ± standard deviations are presented in each experimental condition: horizontal (H0), dorsal flexion at 7° (DF7) and 15° (DF15), and plantar flexion at 7° (PF7) and 15° (PF15). Posturographic parameters are the mean position of the CP along the anterior-posterior (X0) and the medial-lateral (Y0) axes, the mean deviation of the CP along the anterior-posterior (Xm) and the medial-lateral (Ym) axes, and the average velocity of CP (Vm) in the horizontal plane. p-values of the simple contrast analysis, with H0 as the reference condition, are presented. (*p <0.05, **p <0.01, ***p <0.001 and ns = non-significant).

**Fig 8:**
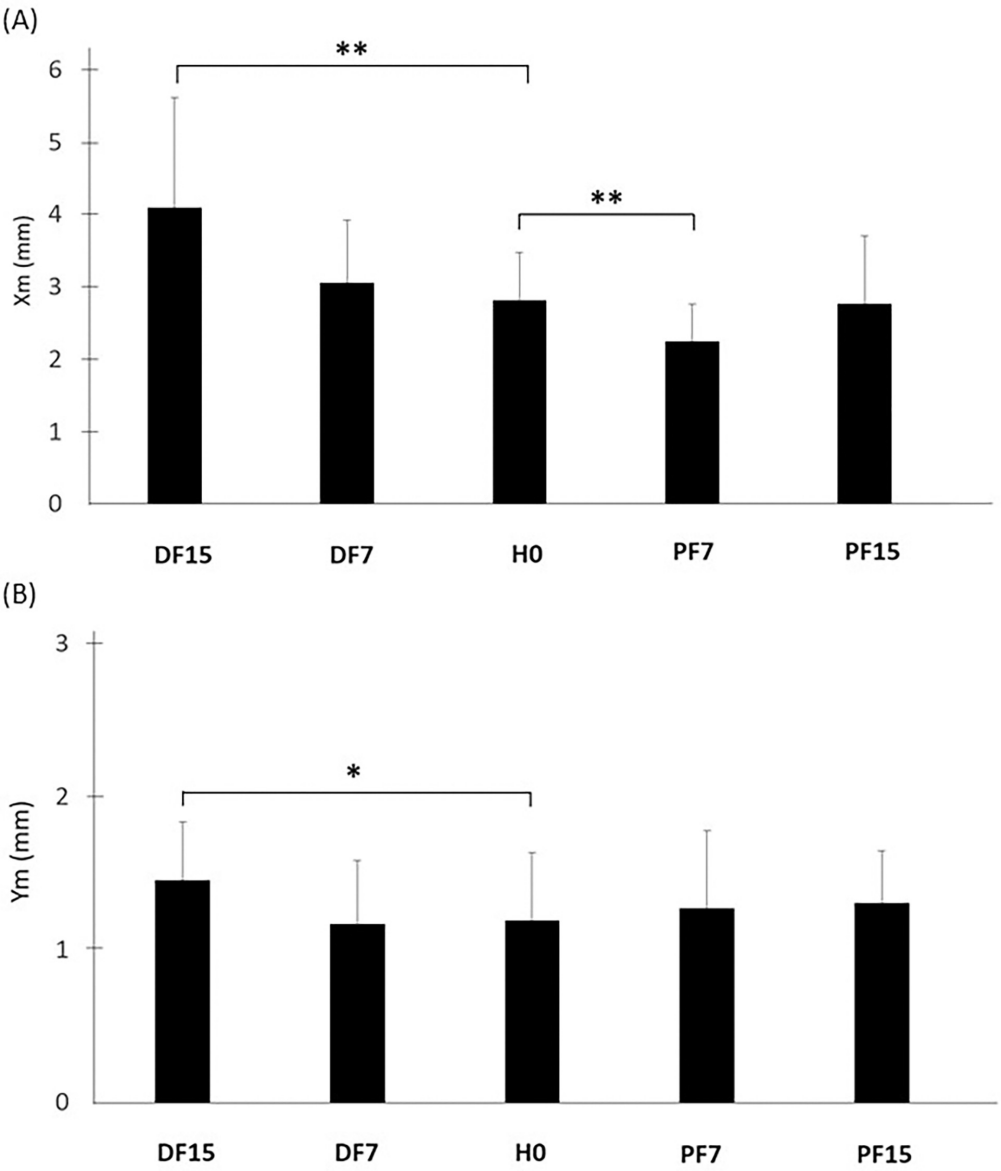
Mean CP displacements along the anterior-posterior (Xm) (A) and medial-lateral (Ym) (B) axes as a function of surface slope. Means and standard deviations are presented in each experimental condition: horizontal (H0), dorsal flexion at 7° (DF7) and 15°, and plantar flexion at 7° (PF7) and 15° (PF15), (*p <0.05 and **p <0.01).

As suggested by raw data inspection, the average displacement of the CP along the anterior-posterior axis (Xm) was also sensitive to support surface slope (p <0.001). However, the contrast analysis revealed that this index increased only for maximum dorsal flexion inclination (DF15) relative to H0 (p <0.01) (Fig 8A and Table 2). Surprisingly, lower values were observed in plantar flexion at 7° (PF7) (p <0.01), while no significant variation occurred in PF15. Along the medial-lateral axis, a global effect also appeared for the Ym index (p <0.05). However, the contrast analysis only revealed a significant difference between H0 and DF15 (p <0.05) (Fig 8B and Table 2).

**Fig 9:**
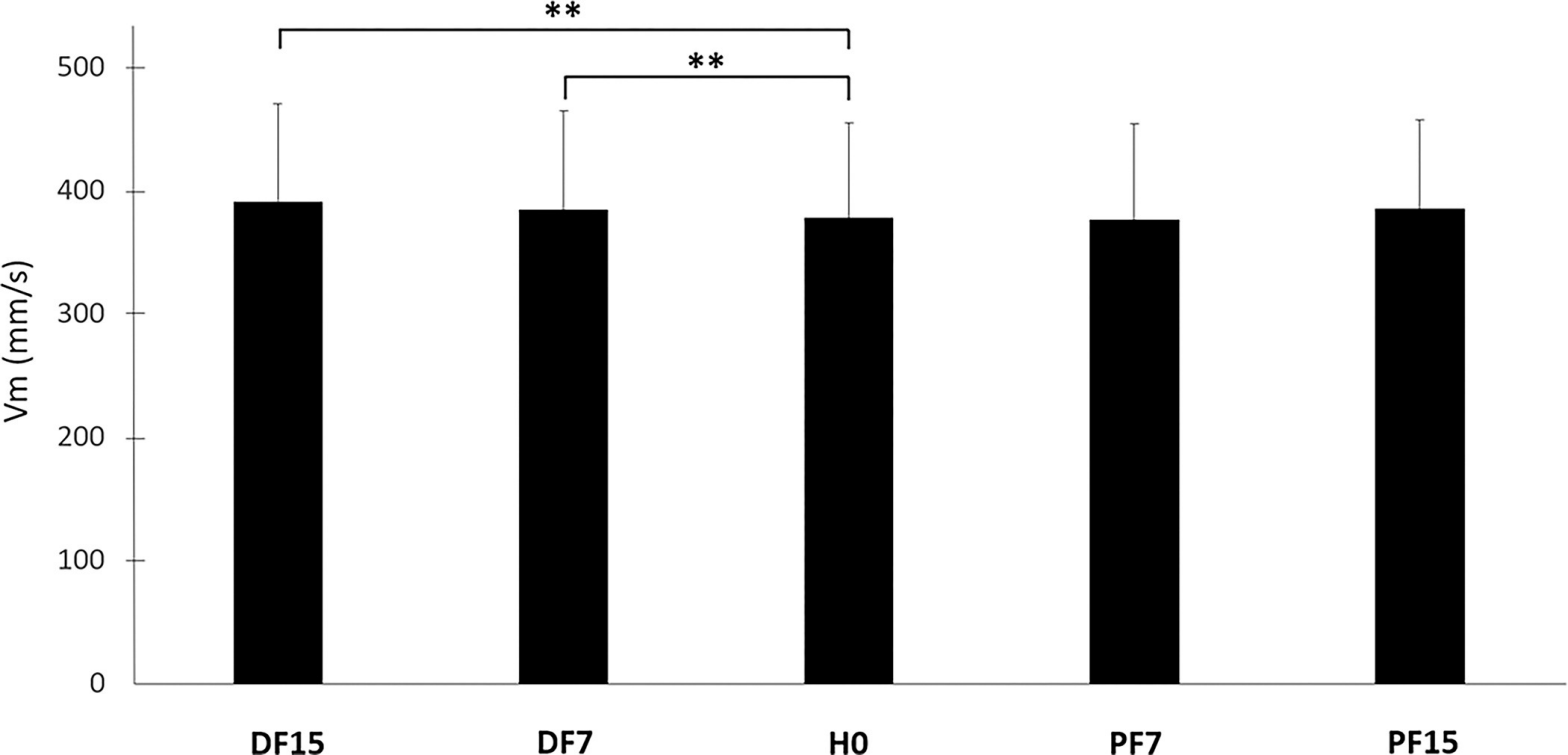
Mean velocity of CP displacements in the horizontal plane (Vm) in each experimental condition. Means and standard deviations are presented for all experimental conditions: horizontal (H0), dorsi flexion at 7° (DF7) and 15°, and plantar flexion at 7° (PF7) and 15° (PF15), (**p < 0.01).

The mean velocity of CP (Vm) also displayed a global effect according to support surface inclination (p <0.05), with higher values in both dorsal flexion conditions (DF7 and DF15 with p <0.01) (Fig 9 and Table 2). The means observed in plantar flexion conditions were very close to those in H0, with no significant variation.

## Discussion

The analysis of posturographic mean position parameters (X0 and Y0) revealed that the CP was shifted in the direction of the surface slope, i.e., forwards for declination (PF7 and PF15) and backwards for inclination (DF7 and DF15). The shift amplitude was also influenced by the angle values, with higher variations at 15° than at 7°. This phenomenon suggests that the slope compensation involving ankle mobility to keep the legs close to the vertical axis was incomplete. This might be related to the passive tension of the soft tissues (muscles, ligaments, and articular capsule) associated with the dorsal or plantar flexion of the ankle, which are likely to pull the legs toward the direction of the slope. In terms of body balance, the shift of the CP towards the inclination direction limits the effective foot base described in the seminal work of Whitney (1962) [22] and, thus, the security margin before falling when facing internal or external disturbances. These results are in line with the study of Gallagher et al. (2013) [23], which found that standing on a declined surface (toes down) causes trunk and lumbar flexion, while an inclined surface (toes up) causes lumbar and trunk extension. However, this differs from the work of Mezzarane and Kohn (2007) [13], who used sloped surfaces at 14° and reported a higher forward lean of the body in the dorsal flexion condition and a smaller one in plantar flexion. We can then consider that for a steeper slope, there is a risk of shifting the CP out of the base of support if pointing in the direction of the slope. The central nervous system (CNS) might then use another strategy, consisting of moving the body in the opposite direction of the slope to prevent the risk of being pulled out of the support base by the tension of stretched soft tissues. In theory, the forward lean should thus involve other joints under the ankle, e.g., the hips or the spine.

Our posturographic data assessing CP displacements revealed that backward sloped surfaces (feet in dorsal flexion) impaired body balance with a significant increase of CP mean deviation along the anterior-posterior axis (Xm, p <0.01 in DF15 relative to H0) and the medial-lateral (Ym, p <0.05 in DF15 relative to H0) axes, as well as that of CP mean velocity (Vm, p <0.01 in DF7 and DF15 relative to H0). Conversely, forward sloped surfaces (feet in plantar flexion) resulted in lower values of Xm when comparing PF7 to H0 (p <0.01), suggesting an improved body balance in PF7. To our knowledge, these results, showing an opposite effect according to the direction of the slope, have not yet been described in the literature. Indeed, most studies show reduced stability when testing both inclined and declined surfaces [8–13] or solely declined [14] and inclined [24] surfaces. However, the slope angles in all these experiments were superior or equal to 10°, focusing on conditions encountered in roof work, while in daily living conditions, such as access ramps and sidewalks, people encounter lower values.

The effect of inclined surfaces on body balance might be related to biomechanical and neurophysiological factors. From a biomechanical point of view, one likely contributing factor is the dorsal flexion of the ankle, which is required to keep the legs close to the vertical axis when standing on an inclined surface. In DF15, the ankle is in a closed pack position [11, 25] because its maximum range of motion is equal to 11.6° to 14.7° in dorsal flexion [18]. It would then require an increased activity of the tibialis anterior to keep that angle against the resistance of the joint’s passive tissues, as attested by significantly higher values of TA EMG in DF15 relative to H0. A closed-pack position and a higher level of muscular activity should thus increase the stiffness of the joint and limit the articular free play needed for the "ankle strategy" used to stabilize the body posture [3].

In addition, the inclination of the support surface reduces the length of the support base, which is equal to the foot length multiplied by the cosine of the sloping angle (trigonometry rule in a right-angled triangle). The CNS must then consider these new mechanical parameters to reprogram the stabilizing mechanisms. This assumption is in line with the view of Simeonov et al. (2003) [14], who declared that the slope surface reduces the effective base of support (i.e., the area of the footprint projection on a horizontal plane) and the safe range of movement of the body’s center of gravity.

From a neurophysiological point of view, one can consider the variation of the somatosensory input related to the inclined surface, as suggested by Simeonov et al. (2003) [14]. Indeed, the shift of the CP toward the heel in DF15 found in our study might impair the accuracy of the information provided by the foot sole to locate the CP, as the mechanical thresholds are higher in the heel area [26]. According to the same study, there is also an increase of afferents from the heel to the toes, suggesting a lower accuracy at the heel level. Moreover, the inclination of the surface backward or forward induces additional shear forces, which might create a mismatch in the interpretation of the mechanoreceptor’s signal intended to locate the CP.

Besides the foot sole, ankle proprioception, specifically muscle spindle activity, is well known for its role in body balance. Experiments using the vibration of Achille tendons, assumed to stimulate muscle spindles [27], resulted in illusory body tilt movements and postural reactions [28]. The stretching of the triceps surae in DF7 and DF15 might, thus, over-stimulate the spindles in unusual static conditions and make interpreting the variations resulting from postural sway more difficult.

The absence of a disturbing effect on body balance associated with plantar flexion in PF15 might be surprising, as it typically induces shear forces on the foot sole and leads to a higher activity of the soleus (Sol) and the gastrocnemius (GasM), which are antagonists of the tibialis anterior (TA). However, the articular free play of the ankle is not as impaired as it is in DF15, as the maximum range of motion is equal to 53.2° to 63.6° in plantar flexion and 11.6° to 14.7° in dorsal flexion [18]. In addition, the increase of Sol and GasM EMG in PF15 relative to H0 is relatively low (+5% for the GasM and +6% for the Sol) compared to the high variation of the TA in DF15 (+15%). Consequently, the risk of limited ankle mobility due to joint or muscular stiffness should be weaker in PF15, with negligible consequences on the ankle strategy involved in postural control [3]. This difference between DF15 and PF15 conditions has been observed in the study of Dutt-Mazumder et al. (2016) [10], which reported greater CP area in toe-up than in toe-down conditions. It must also be noted that nowadays, footwear offers minimal heel support, which mechanically induces plantar flexion, making PF7 a more usual condition for participants.

EMG data showed increased values of the three tested muscles when they were shortened (DF15 for TA, PF15 for GasM, PF7 and PF15 for Sol), and the extensors (GasM and Sol) displayed lower values when they were stretched (DF15). Apart from the higher activity of the TA in DF15, which might be needed to stabilize the joint in a closed pack position, the variation might be related to the muscle length-tension relationship [29]. Based on Hill’s muscle model, this relationship shows that the active tension produced by the sarcomere is consistently lower in the outer and inner ranges of the muscles. In contrast, the passive tension produced by the parallel elastic component increases as a function of muscle length, with maximum values in the outer range. Consequently, in the inner range conditions (DF for TA and PF for Sol and GasM), for which the active part is less efficient, and the passive component displays low tension, the muscular activity level must be high to keep the same level of contraction. Conversely, in outer range conditions, for which the passive tension is very high (DF for Sol and GasM and PF for TA), the level of active contraction needed should be lower.

## Conclusion

Our findings support the idea that backward slope surfaces impair postural equilibrium and require a higher activity of the tibialis anterior to stabilize the ankle joint. Conversely, forward slope surfaces are less likely to affect body balance and could induce a positive effect when set at a small angle. These findings might have an impact in fields such as rehabilitation and accessibility.

## Supporting information

S1 File The normalized EMG and posturographic datas are included. (XLSX)

S2 File (PDF)
