# Supplementary material for: Effect of forward and backward sloped support surfaces on postural equilibrium and ankle muscles activity
Source: PLoS One. 2024 Jun 27;19(6):e0305840. doi: 10.1371/journal.pone.0305840 (PMC11210767; doi:10.1371/journal.pone.0305840)
Supplement: S2 File — (PDF) [file pone.0305840.s002.pdf]

# Ethics approval abstract in english

| |
|---|
| File number: 417<br>Study title : Effect of support surface slope on postural equilibrium and on ankle joint function<br>Responsible of the study: Alain HAMAOUI, full professor<br>Laboratory : CIAMS research unit, Paul Brousse platform<br>Date : june 3rd 2022 |

Decision 1 : **FAVORABLE**

REFERENCE: CER-Paris-Saclay-2022- 045

**Avis donné par les membres du Comité d'Ethique pour la Recherche (CER) de l'Université Paris-Saclay**

Numéro de dossier: 417
Titre de l'étude : Mesure de l'effet de l'inclinaison de la surface d'appui au sol sur l'équilibre postural et la fonction de la cheville
Date de l'étude : réception de l'avis
Demandeur de l'étude : Alain Hamaoui
Date de réception de la demande : 14 avril 2022
Lieu(x) de l'étude : CIAMS Hôpital Paul Brousse
Date d'émission de l'avis : 3 juin 2022
Version d'avis : 2

**Sur proposition des rapporteurs le comité adopte l'avis suivant :**

**Avis 1. Favorable.**

**La référence associée à cet avis est la suivante : CER-Paris-Saclay-2022-045**
